# Considering user experience and cognitive load in the design and development of a virtual reality application for embodied anatomy learning

**DOI:** 10.1002/ase.70319

**Published:** 2026-08-10

**Authors:** Mélanie Gallot, Mira Hajj‐Hassan, Rawad Chaker, Christian Collet, Nady Hoyek

**Affiliations:** ^1^ LIBM, Inter‐University Laboratory of Human Movement Sciences, UR 7424 Universite Claude Bernard Lyon 1 Villeurbanne France; ^2^ ECP Laboratory Université Lumière Lyon 2, ECP Lyon France

**Keywords:** anatomy learning, embodiment, user experience, virtual reality

## Abstract

Virtual reality (VR) offers new possibilities for anatomy education through immersive and embodied interaction. Early‐stage evaluation of VR tools is necessary to assess user experience and perceived cognitive load and to guide iterative development. This pilot study aimed to descriptively evaluate the first prototype of the inCarne VR functional anatomy tool and explore the suitability of an adapted cognitive load questionnaire in this context. Seventeen participants, including kinesiology students and anatomy experts, completed a 15‐min guided VR session involving real‐time body tracking and skeletal mirroring of hip movements. User experience was assessed using the AttrakDiff questionnaire, measuring pragmatic quality, hedonic quality, and attractiveness. Perceived cognitive load was assessed using an adapted French version of the Leppink Cognitive Load Questionnaire. Analyses focused on descriptive statistics, internal consistency, and exploratory correlation coefficients. User experience ratings were descriptively positive across all dimensions, with attractiveness showing the highest mean score (*M* = 1.71, SD = 0.59). Intrinsic cognitive load was reported as low (*M* = 1.54, SD = 1.42), while germane load was higher (*M* = 4.26, SD = 2.95), indicating reported engagement in task‐related cognitive processing. Intrinsic and germane load demonstrated acceptable internal consistency (Cronbach's *α* = 0.834–0.950), whereas extraneous load showed low internal consistency (*α* = 0.283), limiting its interpretability. Exploratory correlations showed moderate to strong descriptive associations between selected user experience and cognitive load dimensions. These findings provide preliminary descriptive evidence supporting the feasibility of the inCarne prototype and inform future development and measurement validation in VR anatomy education.

## INTRODUCTION

### Human anatomy learning and spatial ability

Human anatomy learning is a fundamental part of both clinical professions (i.e., medicine, physiotherapy, or osteopathy) and sport‐based professions (i.e., kinesiology) which require fundamental knowledge of human body structure and functional anatomy comprehension. When learning human anatomy, an underlying difficulty in visualizing the different aspects of the human body causes complexity and abstraction in knowledge gain and understanding.^1^ More precisely, studying the components of interconnected human musculature cannot be achieved through textbooks and traditional lecturing alone^2^: common anatomy failure rates in higher education institutions lead to the withdrawal of students from professional careers. Pujol et al.^3^ believe that 3D interactive models, involving visuospatial abilities, can significantly enhance anatomical education by supplementing current methods. In fact, according to Hoyek et al.,^4^ a large body of research has shown that succeeding in human anatomy learning is related to spatial ability visualization. The complex connections between both have been studied by Guillot et al.,^5^ highlighting the crucial role of individual spatial ability for human anatomy learning. This relationship was shown between anatomy examination results and visuospatial ability scores such as the Vandenberg and Kuse Mental Rotation Test (VMRT).^6^ Spatial ability refers to the ability of a student to identify an object in its different 3D positions after manipulation. Hence, spatial skills are an integral part of anatomy learning, requiring a balance between understanding, memorizing and visualizing.^4^, ^7^ Recent studies show that 3D computer‐based visualization improves anatomy learning outcomes, by enabling the mental representation of structures that leads to knowledge consolidation.^8^, ^9^ Higher achievement in anatomy relies on having a firm understanding of the three‐dimensional relationships among anatomical structures, particularly when viewed from different perspectives.^9^, ^10^, ^11^, ^12^, ^13^ This is consistent with the theoretical association between spatial ability and gross anatomy comprehension.

Visuospatial skills, defined as the ability to mentally process, retain, and manipulate visual information,^14^ are essential for kinesiology students whose future professional tasks involve analyzing complex movement patterns. Radan et al.^15^ found that kinesiology students with higher visuospatial proficiency achieved greater academic success, encouraging their persistence in physical education courses. These findings align with broader research in anatomy and surgery showing that experts outperform novices in visuospatial tasks.^10^, ^16^ Given the importance of spatial ability for professional competency, VR tools could play a significant role in developing students' clinical expertise. Specifically, 3D multimedia tools that train spatial skills such as mental rotation have been shown to improve anatomy learning outcomes for kinesiology students.^4^, ^17^

### Digital technologies for anatomy education

To address students' difficulties with spatial abilities, numerous digital solutions have been developed. A decade of technological development in the paramedical professions has contributed to the advancement of anatomy learning.^18^ In this field, educators' longstanding goal has been to use innovative technology‐based teaching resources, with extended reality (XR) applications being the latest tool currently available.^19^ Higher education institutions are further encouraged to engage in research and development projects to better understand VR efficacy within anatomy curricula.^19^

Previous anatomy applications introduced the 3D technology for education, out of which Visible Body, an@tomedia, Anatomy.app, Complete anatomy (3D4 Medical), Visible Human, Zygote Body, 3D atlas of human anatomy, Anatomy Transfer,^20^ and in our laboratory, Antepulsio.^9^ These applications provided links between human body structures and functional anatomy muscle movement learning, where some of them allow passive information reception (i.e., Visible Body) while some others allow students to interact with the content of the application (i.e., Antepulsio). However, those applications allowed visualization of 3D models in computer‐based settings only with no immersive extended reality visualization via head‐mounted system, nor the possibility of body tracking, helping in functional anatomy learning in particular. Other applications, have allowed anatomical virtual mirrors, helping to reproduce learners' movements in a virtual musculo‐skeletal model for functional anatomy learning (i.e., mirror‐like augmented reality (AR) system,^21^ ARkit human body‐tracking SDK,^22^ magic mirror,^23^ anatomical mirror system^24^). However, these applications allowed an anatomical mirror in augmented reality (AR) settings only. Although those tools provide high real‐time human body tracking and mirroring, the applications allow AR with egocentric experiences only, or a TV screen projection with a motion‐based interface (i.e., magic mirror^23^). Even though these 3D and AR applications have shown a positive impact on learning outcomes, they do not allow the immersion of users in VR learning settings. García‐Robles et al.^25^ showed that VR often shows small‐to‐moderate advantages over non‐immersive 3D, depending on the instructional design and learning context, which may enhance learning outcomes in anatomy education. This is because the immersive nature of VR fosters a more engaging and realistic experience, leading to improved spatial understanding and retention of complex anatomical structures.

### Embodiment and user experience

According to Johnson‐Glenberg,^26^ there are two inherent attributes that make VR a powerful tool for education: (1) the sense of presence and (2) the embodied affordances of gesture and manipulation in 3D space. Indeed, literature stresses the fact that embodiment is a fundamental aspect of the virtual space. It refers to the sense that the learner is inside, possessing, and controlling their virtual projected body in VR. The embodiment afforded by gestures in VR for learning via the hand controllers induces both embodiment and agency, which is the perception that the person is in control of the object. When the content to be learned and the movement performed by the learner are congruent, the similarity between the action performed and the content to be learned can foster learning outcomes.^27^ This congruence was found to be one of the main reasons for the increase in academic results in VR settings.^26^ Embodied cognition was henceforth not only considered a factor in VR development for its relevance in pedagogical applications and results but also for its relation to user experience and design, as shown in the following sections.

Embodiment is a crucial factor in UX design, as it shapes how users perceive and emotionally engage with digital tools.^28^ Elements such as anthropomorphism and bodily cues can foster recognition and empathy through embodied simulations like mimicry,^29^ making them valuable considerations during the design phase.

As incorporating complex user interfaces and information can hinder learning efficiency,^30^ user experience (UX) is henceforth a main goal for educational technology development determining whether the immersive technology is successful.^31^

There are various UX design models applicable to VR applications, each offering distinct advantages; however, the focus will be on agile UX. Agile UX stands out as particularly effective for our study due to its flexibility, iterative focus, and responsiveness to user needs. Agile UX integrates UX practices within agile development methodologies, prioritizing rapid iteration, collaboration, and continuous user feedback qualities essential in VR environments where user interactions are complex and require frequent adjustments to improve usability.^32^ Agile UX supports a dynamic, user‐centered approach that encourages refinement throughout the development process. Agile UX enables an adaptive workflow that addresses learning‐specific needs by incorporating real‐time user feedback, ensuring that the VR application meets end‐user expectations effectively. This approach facilitates a streamlined design process, allowing for ongoing improvements and producing a responsive, engaging, and adaptive VR‐based anatomy learning tool.

In the context of the agile UX methodology, several UX questionnaires are commonly used to evaluate user experiences effectively. The Attrakdiff questionnaire^33^, ^34^ seems the most suitable to answer our research questions described below. In fact, it was used to emphasize its balance between hedonic and pragmatic aspects, which makes it more versatile and comprehensive compared to the other tools.^35^ Hedonic and pragmatic qualities both influence students' attraction to educational tools,^36^ but effective UX design in 3D virtual environments also relies on psychological principles from education and cognition.^37^ Beyond interface design, factors such as instructional strategies and content complexity also contribute to users' cognitive load.^28^, ^38^

### Cognitive load in anatomy learning

To better understand how learners interact with educational VR tools, it is essential to consider the concept of cognitive load, which is central to instructional design and user experience. According to Leppink et al.,^39^ cognitive load can be divided into three types: intrinsic, extraneous, and germane. Intrinsic cognitive load relates to the inherent complexity of anatomical content, while extraneous load stems from how information is presented (such as poor interface design or unclear instructions), which may make the task feel more difficult or frustrating. Germane cognitive load, on the other hand, involves the mental effort invested in understanding and integrating new information. For effective learning to occur, the total of these loads should not exceed the learner's cognitive capacity.^40^ Perceived cognitive load is also shaped by how users experience the task (whether it feels easy or difficult, pleasant, or unpleasant, motivating or discouraging), which directly influences their engagement and overall learning outcomes. While well‐designed VR experiences can support engagement and reduce unnecessary load,^41^ some research has shown that high levels of immersion may increase cognitive demand and potentially hinder academic performance.^42^, ^43^ Moreover, engagement itself can become a source of extraneous load if not properly aligned with the learning objectives,^44^ highlighting the importance of further investigation into how interactivity, perception, and engagement interact in immersive learning environments.^45^

Physical models used in a VR anatomy learning study allowed students to physically rotate the 3D object, which has been suggested to reduce extraneous cognitive load.^46^, ^47^ This potential benefit is supported by literature indicating that well‐designed VR applications can lead to better learning outcomes with less cognitive load.^48^, ^49^ Studies have found that learners' cognitive load negatively affects their intentions to persevere on a task, thereby influencing their UX quality.^50^ To determine whether cognitive load can predict students' UX quality, the final aim of the study is to enhance students' anatomy knowledge and spatial competence after UX agile iterations; therefore, studying the user experience and cognitive load is the primary aim of our study. In addition, this study uses the cognitive load questionnaire developed by Leppink et al.^39^ that was originally validated in a statistical learning context. Hence, the adaptation of the items to our study context is required. When cognitive load instruments are applied outside their original disciplinary or linguistic context, a preliminary evaluation of their suitability is necessary. Consequently, beyond evaluating user experience, the present pilot study also aims to explore the applicability and internal consistency of an adapted version of the cognitive load questionnaire in a VR functional anatomy learning setting. This exploratory approach aligns with established recommendations for pilot testing adapted measurement instruments prior to large‐scale validation or confirmatory studies.

### User profiles and UX perception

Prior research in educational psychology and human‐computer interaction suggests that user expertise influences how interactive systems are perceived and evaluated. According to expertise theory, domain experts and novices differ in how they organize knowledge, allocate attention, and approach problem‐solving tasks.^51^, ^52^ These differences may affect how users experience digital learning tools. From a user experience perspective, Hassenzahl's^53^ model distinguishes between pragmatic quality (perceived usability and task support) and hedonic quality (stimulation and identification).^53^, ^54^ Experienced users may prioritize functional efficiency and goal‐oriented interaction, whereas novice learners may be more sensitive to clarity, guidance, and engagement‐related aspects of the interface. In immersive learning environments, embodied interaction and spatial engagement further shape user experience.^26^ Additionally, cognitive load theory emphasizes that instructional design should consider learners' prior knowledge, as expertise influences how intrinsic and extraneous load are experienced.^39^, ^55^ In VR‐based anatomy learning, expert users (e.g., anatomy teachers) may approach the tool primarily as a functional spatial instrument, whereas novice learners may rely more strongly on intuitive interaction and motivational affordances. Understanding how different user profiles describe their experience is therefore relevant in early‐stage VR development, as it may inform inclusive and iterative design decisions.

### The inCarne prototype

The design of the *inCarne* prototype was informed by three complementary theoretical perspectives: embodied cognition, spatial cognition in anatomy learning, and cognitive load theory. Embodied learning theory suggests that congruence between motor activity and learning content enhances engagement and conceptual understanding. In immersive VR, real‐time movement tracking may strengthen embodiment and presence, which are associated with enhanced learner experience. From a user experience perspective, pragmatic quality and hedonic identification may be positively influenced when learners perceive meaningful alignment between their physical actions and the displayed anatomical content. Cognitive load theory further suggests that well‐structured instructional design should minimize extraneous load while allowing intrinsic and germane load to support meaningful cognitive processing. The minimalist interface and body‐tracked interaction implemented in the *inCarne* prototype were designed to reduce unnecessary interface‐related demands while encouraging active engagement with anatomical structures. Based on these theoretical considerations, the present pilot study explored whether the first prototype would be positively perceived in terms of user experience, whether perceived cognitive load would reflect balanced instructional design, and whether individual differences (e.g., anatomy expertise, VR familiarity) might influence these perceptions.

Building on these empirical premises, this research article is a pilot study, part of an R&D agile UX evaluation of a VR application. Reporting the early stage of evaluation, this pilot study aims to evaluate the UX and cognitive load associated with the first prototype and iteration process between the developers and the target student and expert populations. This prototype involves body tracking and virtual skeletal visualization (the process of creating and interacting with a digital, three‐dimensional representation of anatomical structures) to achieve the final aim of the study consisting in anatomy spatial skill learning.

## AIMS AND RESEARCH QUESTIONS

We aim to validate a first prototype of a research and development project (inCarne), contributing to high‐quality feedback based on user experience and cognitive load. The aim of this pilot study is to test the combination of visualization through an anatomical mirror and visualization to enhance the sense of embodiment via a VR head‐mounted experience.

The research questions (RQ) are the following:

RQ1: How is the first prototype of the *inCarne* VR functional anatomy learning tool evaluated in terms of user experience, as measured by the AttrakDiff questionnaire (pragmatic quality, hedonic quality stimulation, hedonic quality identification, and overall attractiveness)?
RQ2: How are perceived user experience dimensions (AttrakDiff scores) and cognitive load components (intrinsic, extraneous, and germane) measured by the Leppink Cognitive Load Questionnaire in an immersive embodied VR context designed to reduce interface complexity?
RQ3: Do individual differences in functional anatomy expertise (novices vs. experts) and VR familiarity influence user experience (AttrakDiff dimensions) and cognitive load (Leppink Cognitive Load Questionnaire scores) during interaction with the *inCarne* VR prototype?

The results of this current article will serve as the first feedback. They will help with future iterations between teachers and students on one side and developers on the other, to adapt the tool to the target aim and population (first‐year kinesiology students). This process is at the core of our agile development process.

## MATERIALS AND METHODS

### Instructional multimedia tool

The inCarne tool is an innovative instructional multimedia system that leverages virtual reality (VR). Using a VR headset, the tool captures the user's body gestures through a high‐precision camera and reproduces these movements in real‐time within the immersive environment. The gestures are associated with a dynamic skeletal structure, allowing the user to see a skeletal bone representation that mirrors their actions (Figure 2). This integration of real‐world motion with virtual observation provides an intuitive and interactive platform for students to understand functional anatomy movements, as shown in the Figure 1. During the experimental session, participants received verbal instructions describing a predefined list of functional anatomy movements. Participants were asked to listen to each instruction, execute the movement with their own body, and simultaneously observe the corresponding skeletal motion inside the VR headset. The movements were visualized on a full‐body skeletal representation displayed in a 3D virtual environment, enabling users to directly link their physical action to the underlying skeletal motion (Figure 2).

**FIGURE 1:**
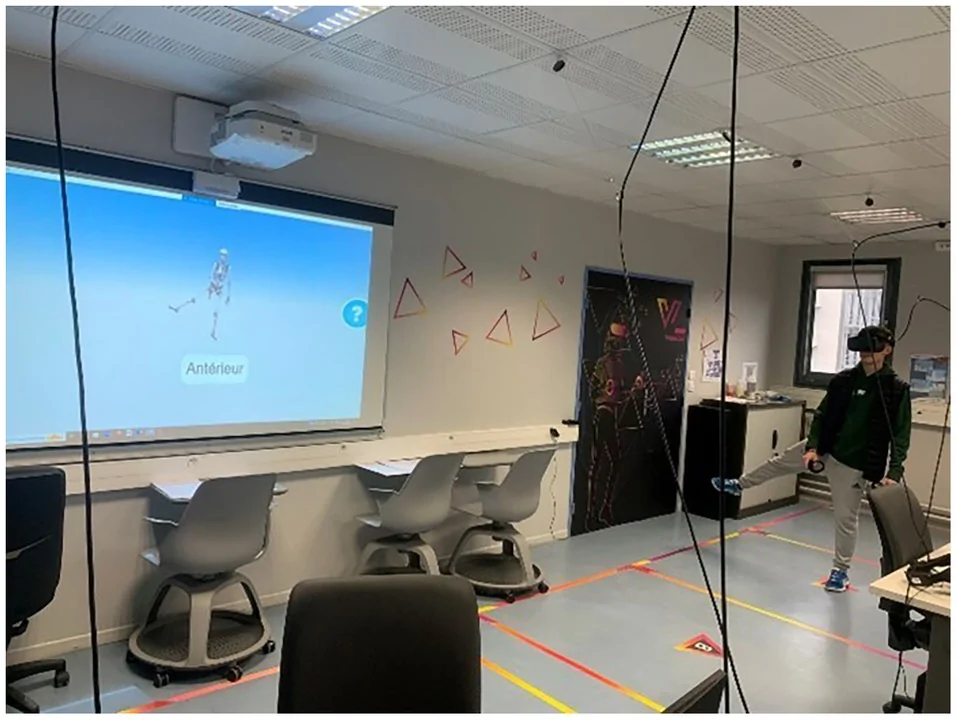
Experimental setup of the inCarne VR study.

**FIGURE 2:**
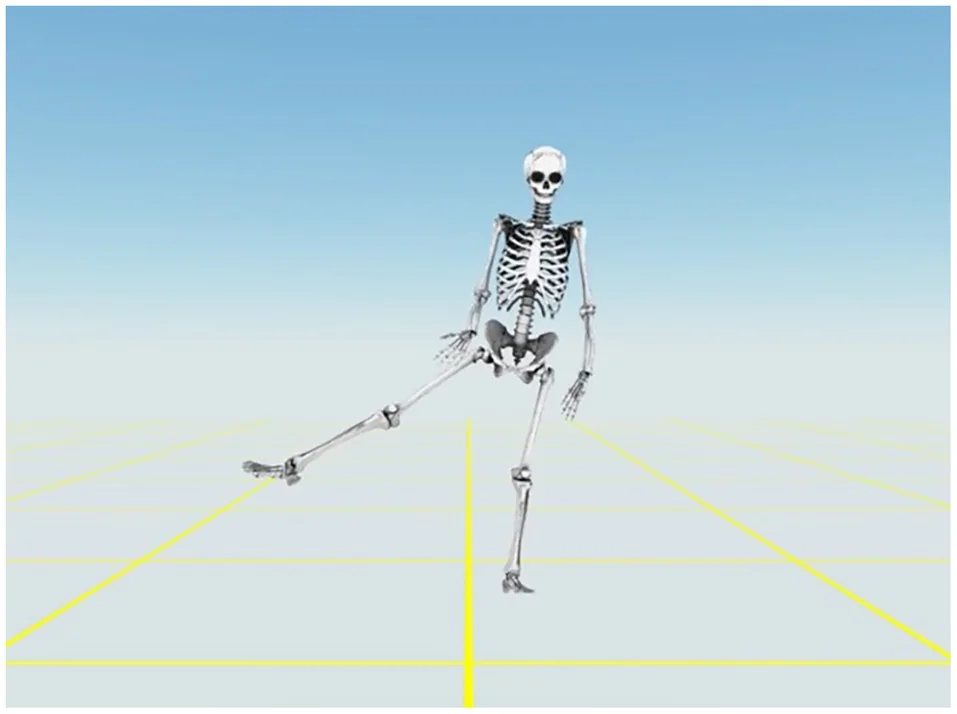
User's view inside the VR headset during the mirroring activity.

Examples of instructed movements included flexion of the thigh on the pelvis (lifting the thigh forward toward the trunk), extension of the thigh on the pelvis (moving the thigh backward relative to the trunk), and pelvic inclination to the right (tilting the pelvis so that the right side moves downward while the left side rises). This coupling between verbal instruction, physical execution, and real‐time skeletal visualization provided an intuitive and embodied approach to understanding functional anatomy, as illustrated by the experimental setup (Figure 1).

The tool allows users to experiment with two activities:

1. Handling activity: Identification and execution of body movements in real time, allowing learners to mobilize their own body while observing the behavior of their anatomical mirror. This activity did not require handheld controllers; movements were tracked by the body‐tracking camera and reproduced directly in the VR environment perceived through the headset. The objective was to promote embodied understanding by directly coupling physical execution with visualized skeletal articulation.
2. Allocentric anatomical spatial orientation: A complementary activity allowing users to manipulate the skeletal model using handheld controllers. Participants used the joysticks to rotate, reposition, and observe the skeleton from different viewpoints. They were instructed to:
  - Rotate the model to obtain anterior, posterior, medial, and lateral views,
  - Position the model in combined spatial orientations (e.g., antero‐superior, postero‐lateral),
  - Zoom in on the hip joint region to inspect spatial relationships between structures.

Before each manipulation task, participants were instructed to return to the anatomical reference position to maintain consistency.

The tasks were designed to support the following intended learning outcomes:

1. Improve spatial understanding of hip joint orientation and anatomical reference positions.
2. Facilitate comprehension of the relationship between joint movement and skeletal articulation.
3. Support integration of motor execution with structural visualization in functional anatomy.
4. Enhance three‐dimensional mental representation of anatomical structures.

The pilot study did not aim to measure learning gains directly but to evaluate whether the interaction design and task structure were perceived as usable and cognitively manageable within this embodied learning context.

### Design features evaluated in the inCarne prototype

The first prototype of the *inCarne* application was intentionally developed around specific instructional and interaction design principles that were evaluated during this pilot study. The following core design features were implemented and evaluated:

1. Real‐time body‐tracked skeletal mirroring (embodiment controllers)
  The system captures full‐body movements through a motion‐tracking camera and reproduces them in real time via a skeletal avatar displayed in the VR headset. This design enables learners to execute anatomical movements with their own body while simultaneously observing the corresponding skeletal articulation. No handheld controllers are required in this mode, promoting direct embodied interaction.
2. Body content congruence
  Functional anatomy movements (e.g., hip flexion, extension, abduction, adduction) were verbally instructed and physically executed by participants. The skeletal avatar mirrored these actions in real time. This design was intended to align motor execution with anatomical visualization, supporting embodied learning through congruence between action and content.
3. Dual spatial reference frames (egocentric and allocentric perspectives)
  The prototype integrates two complementary spatial cognition modes:
  - An egocentric embodied mode, where users observe their own skeletal representation while moving.
  - An allocentric analytical mode, where users manipulate and rotate the skeletal model using handheld controllers to inspect anatomical structures from multiple viewpoints.
  This dual‐frame structure was designed to support both experiential understanding (through bodily movement) and structural analysis (through external observation).
4. Minimalist interface to reduce extraneous load
  The interface was intentionally designed without complex overlays, menus, or excessive graphical elements. The aim was to limit visual clutter and reduce potential sources of extraneous cognitive load during the immersive experience.
5. Zoom and regional focus capability
  Users could zoom in on specific anatomical regions during allocentric manipulation. This feature was intended to support spatial exploration and targeted structural analysis without overwhelming the learner.

These design features were collectively evaluated through user experience and perceived cognitive load measures to assess their suitability and practical feasibility in an immersive functional anatomy context.

### R&D process for developing the inCarne tool

An agile software development method was used to design inCarne application. The objective of this process was to iteratively develop and refine a functional anatomy learning prototype based on early user feedback and usability observations. Based on prior research and learning principles, students' success factors in anatomy learning were first identified. The interactive digital tool was then conceived and refined through iterative feedback collected from novice users (learners without functional anatomy expertise) and expert users (functional anatomy teachers).

This study was conducted with different partners, academic, and industrial. Academic partners provided the inCarne skeleton 3D model as it is a fundamental component to ensure the quality of anatomical accuracy, such as the spatial relationships between each structure.

The model was thus cleaned and technically standardized by industrial partners and adapted to use in the inCarne interactive application. In parallel, the partner company developed the tool using the body‐tracking motion capture camera (Zed explorer Z2i), enhancing it with several movement capture, analysis, and functionalities. The body‐tracking camera was selected for its ease of deployment, as it does not require wearable trackers and for its low cost, making it suitable for scalable and practical implementation in educational settings.

The application offers two spatial cognition frames of references: (i) an egocentric view supported by the body‐tracking feature, allowing users to observe their movements and positions without controllers, and (ii) an allocentric spatial orientation, allowing users to rotate and control the 3D model of their own mirror anatomy to see the model from all angles in a specific position using controllers. In addition, the tool enables users to focus on specific anatomical regions by zooming in and out of the model using controllers. At the application‐use level, user experience revealed a limited field of view, as the skeleton could not be seen when the user turned their body in an egocentric setting without controllers.

### Sample

This pilot study employed a quantitative, descriptive design to explore user experience and perceived cognitive load during interaction with a virtual reality functional anatomy learning tool. The sampling method followed in this article was non‐random. A sampling technique can be classified as probability (based on random criteria) or non‐probability (based on predefined criteria), according to Elfil and Negida.^56^ The study targeted first‐year kinesiology students and anatomy teachers. A non‐random snowball sampling method was used: an invitation email was sent via a university mailing list, and recipients were encouraged to forward the invitation to colleagues who met the eligibility criteria. Participation was voluntary, and all participants were informed about the study objectives, confidentiality, and anonymity. Data were stored on secure university servers in compliance with GDPR regulations (General Data Protection Regulation). As the study involved non‐interventional pedagogical activities, the local human research committee of Lyon 1 University granted ethical approval. The final sample consisted of a total of *N* = 17 participants (76.5% male, age: *M* = 23.53, SD = 6.6 years).

The survey used for data collection employed a questionnaire measuring items related to individual characteristics (Table 1). The functional anatomy knowledge level was evaluated based on participant status. The novice participants included first‐year kinesiology students, whereas the expert participants included anatomy teachers.

**TABLE 1:** Sample characteristics.

Variable	Modality	Percent (%)	Frequency
Gender	Male	76.5	13
	Female	23.5	4
Hand dominance	Right‐handed	100	17
Functional anatomy knowledge level	Expert	41.2	7
	Novice	58.8	10
VR familiarity	Familiar	52.9	9
	Not familiar	47.1	8

### Experimental design

The kinetic movements chosen for this study focused on the hips. The training session lasted 15 min for each participant, conducted individually. No participant withdrew from the testing experience. Prior to the experience, the participants were invited to try the controllers' functionality and get familiar with the hardware design. This task aimed to help participants become familiar with the manipulation of the skeleton and its positioning using controllers (zoom in/out, rotate the skeleton in different directions, move it up/down, shift it left and right). Furthermore, participants were invited to familiarize themselves with their anatomical skeleton mirror without using the controllers (moving in all directions and observing the skeleton mimic their movements).

After the familiarization stage, all the participants performed the two activities allowed by the tool:

1. Handling activity: Body positioning evaluations were the following: posterior, anterior, medial, and lateral view of the right hip, hip flexion and extension, hip abduction and adduction.
2. Allocentric anatomical spatial orientation using controllers in both hands: This practice exercise involved using the joysticks to orient the skeleton's right hip in different simple views as posterior, anterior, lateral, medial, and combined views as antero‐superior, antero‐medial, postero‐lateral for example. During this training, participants were asked before the execution of each position to get back in front of the camera in the anatomical reference position.

Afterwards, participants were invited to answer paper‐based surveys presented in the measures section. Moreover, participants were also asked to provide qualitative feedback on their user experience, suggesting points for improvement on the current version as well as their overall thoughts about the tool.

### Measures

Several survey questionnaires were employed to evaluate different components of user experience:

Cognitive load questionnaire^39^: It is designed to assess the mental effort and resources required by a user during a learning or task‐oriented activity and rated on a 10‐point Likert scale. The survey uses nine items to quantify diverse types of cognitive load, helping to understand how task design influences mental processing. Each type of cognitive load (i.e., intrinsic load (IL), extraneous load (EL), and germane load (GL), in the literature) is composed of 3 items. In the present study, the questionnaire was administered in French. As no fully validated French version was available and the original instrument was developed in a statistical learning context, minor wording adaptations were made to ensure clarity and relevance for an anatomical education setting, while preserving the theoretical constructs underlying intrinsic, extraneous, and germane cognitive load. Given these adaptations, the questionnaire was intentionally used as part of this pilot study to explore the suitability, clarity, and internal consistency of the adapted items rather than as a fully validated outcome measure. Internal consistency analyses were therefore conducted to examine the reliability of each cognitive load dimension in this context.

Attrakdiff questionnaire^33^, ^34^ was used to measure participants' user experience. This questionnaire is made up of 25 items. These items are presented as bipolar adjective pairs, rated on a 7‐point scale. The 25 items are grouped into four dimensions: (1) Pragmatic quality (7 items), which measures the usability of the product. A high score means that the product is considered effective, functional, and easy to use. (2) Hedonic quality simulation (6 items), which measures the stimulation generated by the system. A high score suggests that users perceive the product as aesthetically pleasing and evoking positive emotions and not being boring. (3) Hedonic quality identification (6 items), which measures the user's identification with the system. A high score suggests that the product meets their needs, reflects their identity, or offers personalization options that enhance their engagement. (4) Attractivity (6 items), which measures the overall attractiveness of the system, based on pragmatic and hedonic qualities. A high attractiveness score indicates that the product is perceived as attractive, engaging, and desirable and generates user interest.

### Data analysis

Data were analyzed using descriptive statistical methods with SPSS Version 29.0. Analyses focused on summarizing user experience scores, perceived cognitive load levels, and internal consistency of questionnaire dimensions. To explore associations between user experience dimensions and perceived cognitive load components, Pearson correlation coefficients were calculated and summarized in correlation matrices. In the context of this descriptive pilot study, correlations were used to examine the direction and strength of linear relationships between variables rather than to evaluate confirmatory hypotheses. For the Pearson coefficient, a score of +1 means a perfect positive relationship whereas a coefficient of 0.8 indicates a strong positive relationship.^57^

The convergent validity of the model was studied through Cronbach's alpha values. As a measure of scale reliability, Cronbach's alpha measures the degree to which items are closely related within a set of items. Therefore, the convergent validity of the model was studied through Cronbach's alpha values. Previous studies have suggested values of 0.7 and above for Cronbach's alpha; this indicates that the construct used for quantifying the variable is consistent.^58^

## RESULTS

### Validity and reliability scores

According to the Shapiro–Wilk test, the data showed normal distributions for the factors (Table 2 Normality column). Several scores were calculated to assess the survey's validity and reliability (Table 2):

**TABLE 2:** Validity and reliability scores.

Survey	Factors	Mean	SD	Skewness	Kurtosis	Cronbach alpha	Normality
UX (Attrakdiff)	Pragmatic quality	0.95	0.85	−1.4	4.39	0.726	0.870
	Simulation hedonic quality	1.56	0.80	−0.37	−0.50	0.854	0.959
	Identification hedonic quality	0.86	0.82	0.90	1.48	0.648	0.940
	Attractivity	1.71	0.59	−0.54	0.92	0.830	0.955
Cognitive load	Intrinsic Load	1.54	1.42	1.54	3.4	0.834	0.866
	Extraneous load	1.17	1.24	1.23	1.11	0.283	0.860
	Germane load	4.26	2.95	−0.229	−1.25	0.950	0.920

#### Research question 1: Feedback on the first inCarne tool version

Following the Attrakdiff user experience reporting method, the score of all the participants suggests that the prototype of the product is situated between the self‐oriented and desired areas (Figure 3, see gray dot).

**FIGURE 3:**
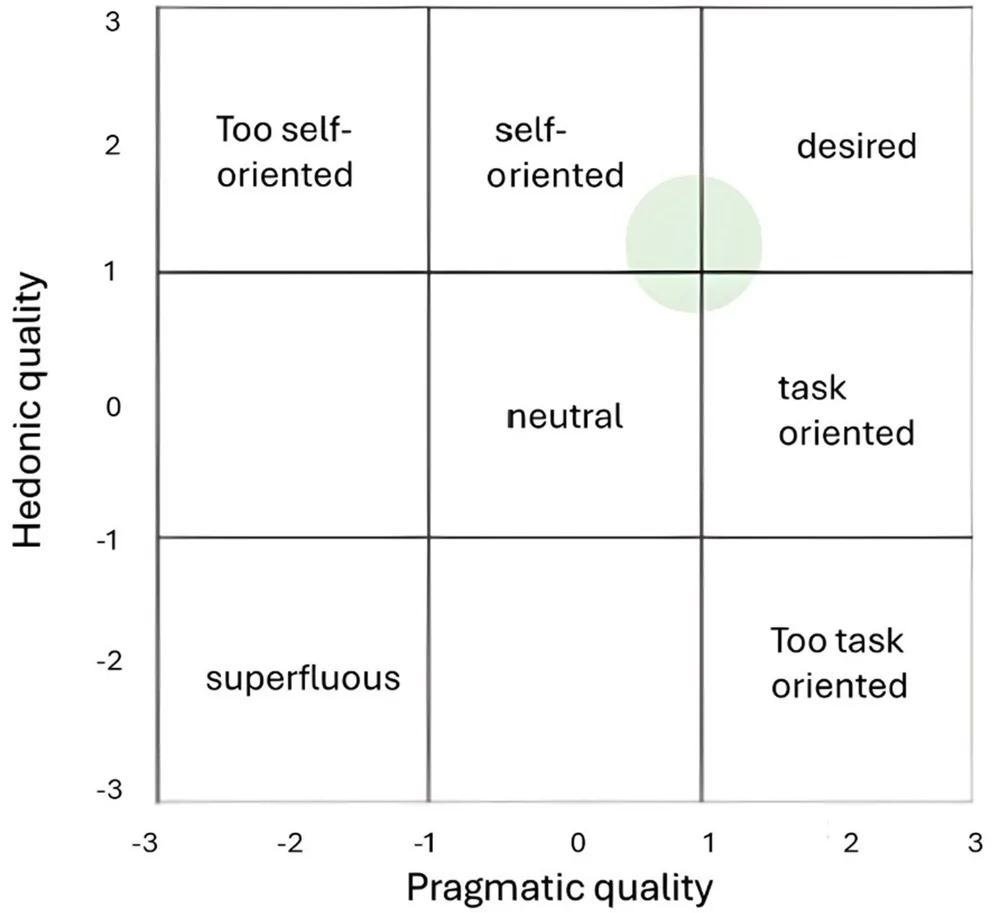
User experience results of Attrakdiff dimensions.

Attractivity (ATT) is the strongest performing dimension, reflecting users' high satisfaction or positive attitudes. Identification hedonic quality (QHI) scored the lowest but remains above 0, however, having marginal consistency, no clear outcome about this factor indicates the QHI's exact representation. The overall diagram is positive, with all scores above 0, showing general satisfaction with the tool's user experience (Figure 4).

**FIGURE 4:**
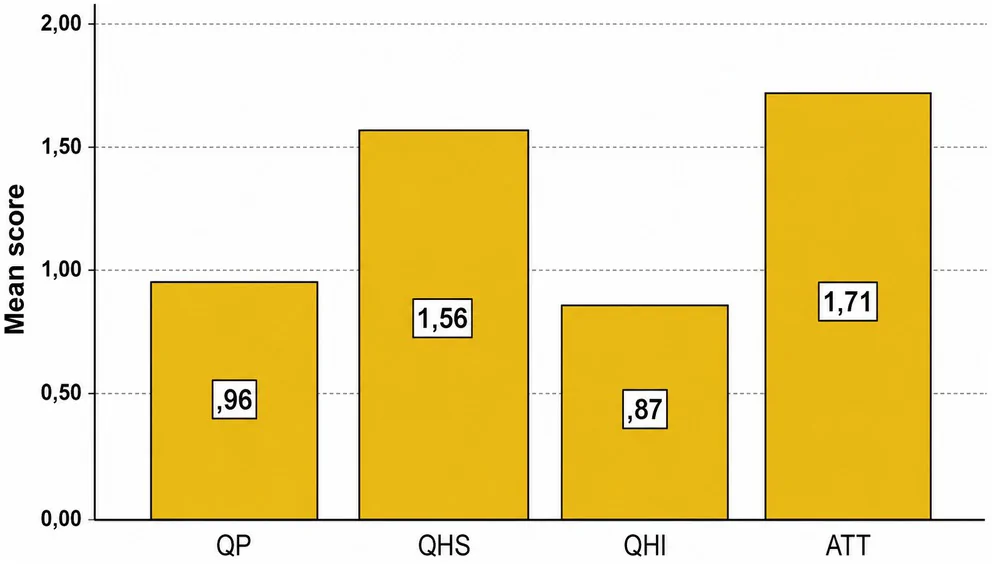
Mean value of Attrakdiff dimensions. ATT, attractivity; QHI, identification hedonic quality; QHS, simulation hedonic quality; QP, pragmatic quality.

#### Research question 2: Interaction between user experience and cognitive load

##### Factors correlation

Pearson correlation coefficients were calculated to descriptively explore associations between user experience dimensions and perceived cognitive load components (Table 3). Numbers 1 to 4 indicate Attrakdiff dimensions and numbers 5 and 6 indicate cognitive load factors. The better correlation is observed between the Intrinsic load (IL) and the Germane load (GL) (*r* = 0.661).

**TABLE 3:** Pearson correlation between Attrakdiff and cognitive load factors surveys.

		1	2	3	4	5
1	QP	—				
2	QHS	−189	—			
3	QHI	0.416	0.207	—		
4	ATT	0.349	0.202	0.492	—	
5	IL	−0.159	−0.139	0.062	−0.230	—
6	GL	−0.046	0.007	−0.013	0.059	0.661

Abbreviations: ATT, attractivity; GL, germane load; IL, intrinsic load; QHI, identification hedonic quality; QHS, simulation hedonic quality; QP, pragmatic quality.

#### Research question 3: Individual differences predictors

To answer RQ3, descriptive statistics showing the means (M) and standard deviation (SD) for each group difference category were calculated. We compared cognitive load and UX measures according to functional anatomy knowledge level (Experts vs. Novices), participants' gender (Female, Male), and according to VR familiarity level (familiar vs. not familiar) as shown in (Table 4).

**TABLE 4:** Comparative descriptive table between factors and individual differences.

Factors	Anatomy knowledge level				Gender				Familiarity with VR			
	Expert		Novice		Female		Male		Yes		No	
	*M*	SD	*M*	SD	*M*	SD	*M*	SD	*M*	SD	*M*	SD
QP	0.59	1.03	1.21	0.62	0.17	1.23	1.19	0.56	1.12	0.64	0.76	1.05
QHS	1.53	0.54	1.58	0.96	1.57	0.50	1.56	0.88	1.71	0.58	1.39	1.01
QHI	0.32	0.38	1.24	0.85	0.82	0.44	0.87	0.92	1.15	0.89	0.53	0.63
ATT	1.36	0.53	1.95	0.51	1.60	0.45	1.74	0.63	1.71	0.72	1.71	0.43
IL	1.19	0.99	1.80	1.66	2.75	2.23	1.17	0.91	1.77	1.61	1.29	1.22
EL	1.52	1.59	0.93	0.94	1.75	0.83	1	1.31	1.11	1.40	1.25	1.12
GL	2.92	3.22	5.2	2.49	5.37	3.85	3.92	2.71	4.47	2.57	4.03	3.50

Abbreviations: ATT, attractivity; EL, extraneous load; GL, germane load; IL, intrinsic load; QHI, identification hedonic quality; QHS, simulation hedonic quality; QP, pragmatic quality.

Mean pragmatic quality scores were also comparable between participants familiar with VR and those without prior VR experience. Concerning gender, mean pragmatic quality scores were higher for males. Moreover, overall attractiveness ratings were identical across groups (Table 4).

Concerning participants' score differences in terms of functional anatomy expertise, novice participants reported higher scores than experts on hedonic quality identification (QHI) (*M* = 1.24; SD = 0.85 vs. *M* = 0.32; SD = 0.38) and on attractivity (ATT) (*M* = 1.95; SD = 0.51 vs. *M* = 1.36; SD = 0.53, Figure 5).

**FIGURE 5:**
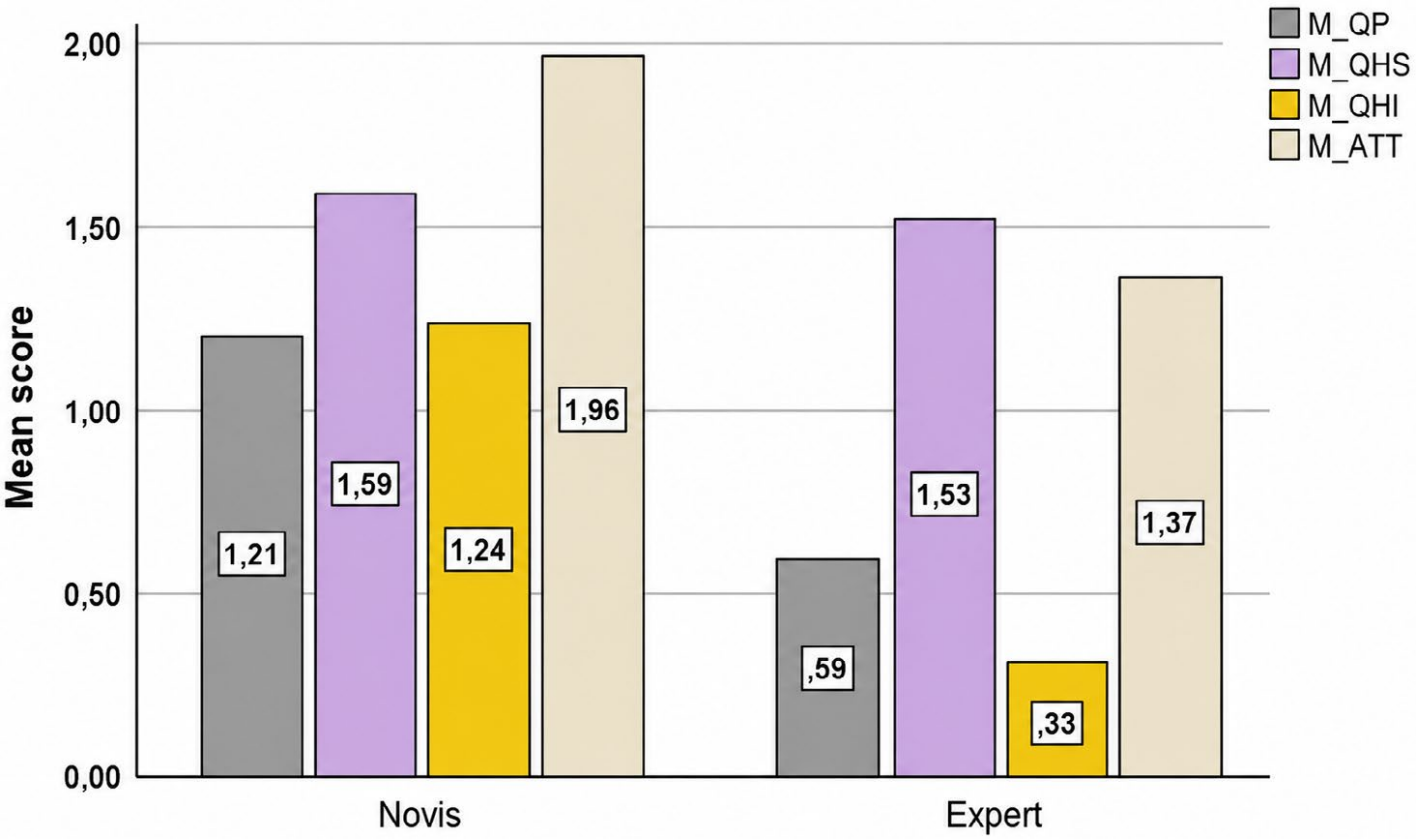
Mean value of the Attrakdiff survey of both experts and novices groups.

Following the Attrakdiff user experience reporting method (Figure 6), the experts' dot is located along the horizontal axis. However, the vertical position of the expert's blue dot is closer to the neutral quadrant. Concerning the novices, their dot is slightly advancing the blue dot on the horizontal axis. However, the vertical position is located higher on the vertical axis (positive quadrant). Experts perceived the tool to be more task‐oriented and neutral compared to the student participants, who found the tool to be more desired and self‐oriented. This difference was also underlined in the average user experience differences between experts and novices. In fact, students were more positive about the tool use pragmatic quality, identification hedonic quality, as well as about the attractiveness dimension of the tool (Figure 5).

**FIGURE 6:**
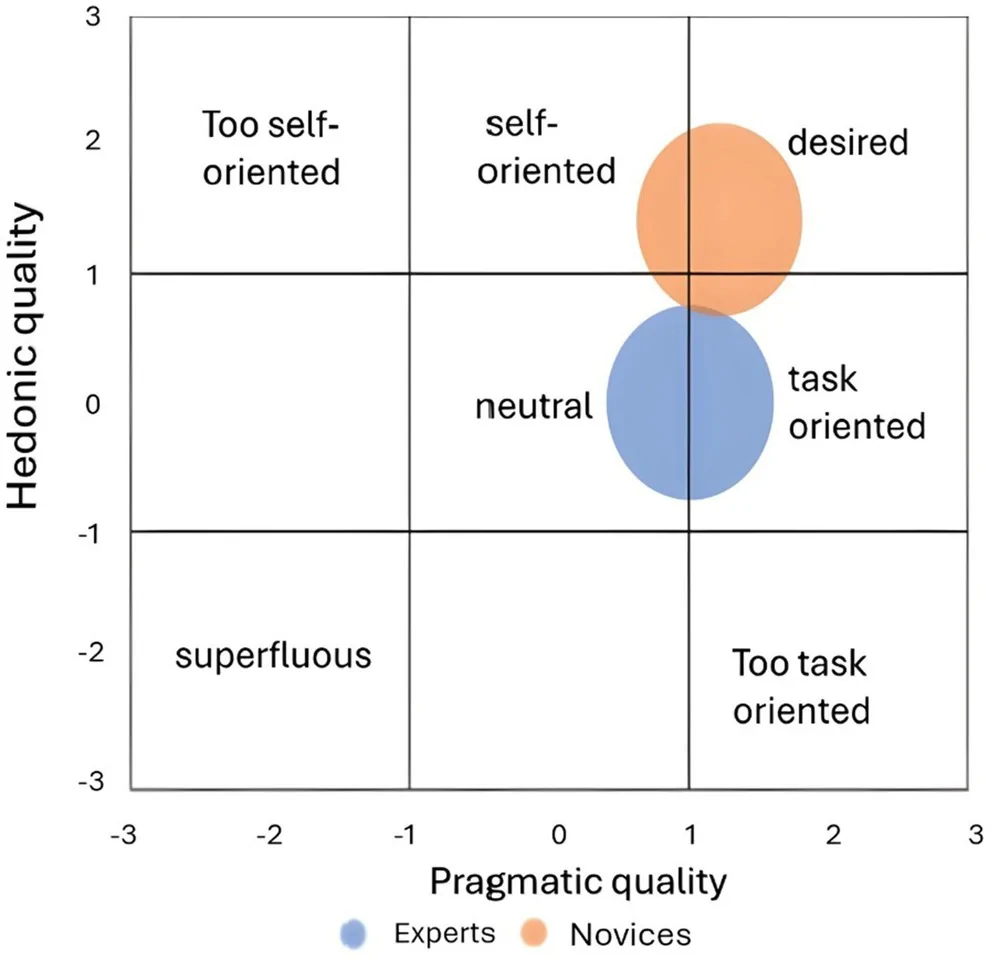
User experience results of Attrakdiff survey of both Experts (blue) and Novices (orange) groups.

## DISCUSSION

This pilot study aims to analyze the user experience of the inCarne VR functional anatomy learning tool, uncovering the individual factors influencing the immersive experience, in particular individual characteristics and cognitive load. Thus, this article aims to answer the following research questions: RQ1: “How is the first prototype of the *inCarne* VR functional anatomy learning tool evaluated in terms of user experience, as measured by the AttrakDiff questionnaire (pragmatic quality, hedonic quality stimulation, hedonic quality identification, and overall attractiveness)?” RQ2: “How are perceived user experience dimensions (AttrakDiff scores) and cognitive load components (intrinsic, extraneous, and germane) measured by the Leppink Cognitive Load Questionnaire distributed in an immersive embodied VR context designed to reduce interface complexity?” and RQ3: “Do individual differences in functional anatomy expertise (novices vs. experts) and VR familiarity influence user experience (AttrakDiff dimensions) and cognitive load (Leppink Cognitive Load Questionnaire scores) during interaction with the *inCarne* VR prototype?”

### Feedback on early‐stage incarne tool user experience

The inCarne tool was positively perceived by all the participants in its first version. In fact, the average user experience score was above the neutral score line in the diagram of mean values showing an overall satisfaction with its use. Additionally, user experience results revealed that the tool is self‐oriented and desired, thus making users think about their own educational needs and experiences rather than the needs of the application itself, which could be further optimized. The positive value along the horizontal axis indicates that the results prioritize usability and task efficiency. Users can complete their tasks effectively, and the solutions are functional and intuitive; the slightly positive position on the vertical axis suggests that the results also incorporate enjoyable or emotionally engaging elements, though not overwhelmingly so. Users find the design appealing, which adds to their satisfaction.

Descriptive results indicated that attractiveness (ATT) was the strongest performing dimension whereas identification hedonic quality (HQI) scored the lowest. Higher identification hedonic quality scores descriptively reflected participants' subjective appreciation and engagement with the system's experiential characteristics. The results highlighted potential areas for refinement in future iterative development. Descriptive correlation analysis showed a moderate association between pragmatic quality (QP) and attractiveness (ATT) ratings of the anatomy VR learning software in this pilot sample. This aligns with the findings of Schrepp,^59^ which indicated that pragmatic quality has a greater influence on attractiveness than hedonic qualities in business management software. Furthermore, the sum of pragmatic quality and hedonic qualities (identification and stimulation) correlated with ATT, aligning descriptively with the theoretical framework proposed by Hassenzahl^36^ as described in the literature and aligning with the findings of Chaker et al.^35^

The subgroups patterns (expertise, VR familiarity and gender) indicated relevant results for this first prototype. In regards to participants' differences in terms of functional anatomy expertise, both groups appeared able to use the system successfully and reported a generally positive experience, which supports the usability of the prototype across different levels of prior domain knowledge. Results showed that students rated the user experience of the inCarne tool better than experts. In fact, experts found the immersion less satisfying than novices and indicated areas where the application might improve to better meet the expectations of experienced users, such as deeper immersion or advanced features. However, our results showed that lower anatomy knowledge may lead participants to perceive VR as a more effective or engaging tool for functional anatomy training compared to those with greater knowledge. Indeed, the expectations that college students have for digital technology in their coursework are significantly shaped by the technologies they have previously used in their personal lifes.^60^ For these users, the embodied and spatial features of VR may provide added value by making complex anatomical content more concrete, interactive, and meaningful. By contrast, experts may have evaluated the system from a more critical or instrumental perspective, drawing on prior disciplinary knowledge and established strategies.

Descriptive comparisons based on participants' familiarity with VR did not reveal consistent or substantial impact on participants' overall experience of the inCarne prototype. In particular, no clear differences across user experience or perceived cognitive load dimensions were clearly identified (Table 4). These findings suggest that the prototype was approachable by both VR experienced participants and those who are not. Concerning genders' comparisons, males scored better than females for the pragmatic quality dimensions. However, this is a preliminary result, largely due to the small overall sample size and the limited number of participants within each gender category.

### Cognitive load measurement

In our study, the relatively low mean of intrinsic load indicates that the tasks involved in the process are not inherently complex or cognitively demanding for the users. This low intrinsic load is positive in scenarios where simplifying complex concepts is a goal. It implies that the users can focus on other aspects, such as usability or deeper engagement. The mean extraneous load scores were descriptively low, which should indicate that unnecessary distractions or poorly designed interface elements are minimal; however, this dimension demonstrated low internal consistency, and the extraneous dimension should be re‐measured in the main study. This could reflect an efficient design where users can concentrate on the core tasks without being overwhelmed by irrelevant information or suboptimal design. The low SD suggests consistency in user experiences across participants. The germane load is higher (Mean = 4.26), with a greater variability (SD = 2.95). Germane load scores were descriptively higher, suggesting that participants reported engagement in task‐related cognitive processing during interaction with the system. The relatively high SD suggests differing levels of engagement or expertise among participants. High germane load is generally positive, especially in scenarios focused on active learning, problem solving, or deeper engagement.^61^ However, the variability might point to inconsistencies in how users interact with the system. These exploratory findings provide preliminary insight into how participants described their cognitive workload when interacting with the inCarne prototype and may inform future iterative development and evaluation. The most notable association was observed between intrinsic load (IL) and germane load (GL), which showed a positive correlation (*r* = 0.661), indicating that higher perceived task‐related complexity was accompanied by higher reported engagement in meaningful processing.

In addition to examining user experience, this pilot study provided valuable insights into the behavior of an adapted cognitive load questionnaire in a VR anatomy learning context. The intrinsic and germane load dimensions demonstrated acceptable to high internal consistency (Cronbach's *α* equal to 0.834 and 0.950, respectively), suggesting that these constructs were well understood by participants and coherently measured in this setting. These values are comparable to those reported in the original instrument^39^ and support the relevance of these dimensions for VR‐based anatomy learning. In contrast, the extraneous load dimension showed poor internal consistency, with negative or low Cronbach's alpha values. Item‐level analyses indicated that the extraneous load items may have been either inadequately adapted linguistically or insufficiently aligned with the specific characteristics of the VR task. This finding suggests that extraneous load, as operationalized in the adapted questionnaire, did not function reliably in this pilot study. Importantly, identifying such measurement limitations is a key objective of pilot research. These results highlight the need for further refinement or replacement of extraneous load items when assessing cognitive load in immersive VR anatomy learning environments.

Moreover, results illustrate the distribution of perceived cognitive load components within this VR interaction context. Debue et al.^62^ found that germane and extraneous loads exhibited opposite relationships, that germane load and cognitive absorption exhibited a positive association, and that intrinsic and germane loads had a non‐linear relationship. These results were confirmed by Kok et al.^63^ in their recent findings. A moderate positive association was observed between intrinsic and germane load in this pilot sample. In addition, results show that users reported low overall intrinsic cognitive load, and a high score for germane load. Schnotz and Kürschner^64^ claim that learning can occur without germane load, but it can enhance learning as it results from instruction and contributions that are beneficial for learning. Extraneous cognitive load generated by the way the material is presented, which does not aid learning, cannot be clearly identified in this study due to the scale's inconsistent reliability. Our results are consistent with Khalil^65^ in terms of GL and IL, who found it important to maximize germane load, to optimize intrinsic load, and to minimize extraneous load for optimal learning.

Attractiveness, in this context, reflects the overall perceived value of the product, encompassing its pragmatic and hedonic qualities, as found in our results, which confirm those of Hassenzahl^36^ previously in the literature. This pattern suggests that the cognitive load introduced by the design of the tool may be associated with its attractiveness. In this study, participants perceived the tool as less cognitively demanding, alongside a manageable level of appeal, thus nuancing the role of reducing cognitive load in making a product more attractive to users. However, our study did not reveal a clear negative relationship between overall cognitive load and user experience. This lack of clarity may be attributed to the small sample size used in the study and the low EL Cronbach's alpha. Further research with a larger sample is necessary to better understand and establish more definitive relationships between these factors. Nevertheless, cognitive load and user experience in VR were found to be associated in this dataset. Prior literature suggests that VR interface design may influence perceived cognitive workload by presenting information in a clear and intuitive way, which helps students better understand and retain concepts without becoming cognitively overloaded.^66^ User experience encompasses ease of use, interface intuitiveness, and user enjoyment. An improved VR user experience can foster better engagement and reduce cognitive load related to the task difficulty by making learning more accessible and enjoyable.^66^

Concerning the QP and QHS of user experience as well as the IL, GL cognitive load factors, no consistent descriptive differences were observed, suggesting that anatomy knowledge does not directly affect the overall quality of the VR presentation, interaction with the system, or cognitive load levels. These results are in line with those of Michalski et al.^67^ stating that mixed reality applications can enhance classroom and laboratory anatomical instruction without imposing excessive cognitive load on learners. However, students qualitatively reported finding the controllers harder to use than experts and more cognitively demanding. Indeed, “Cognitive load theory assumes that the higher the learner's prior knowledge (i.e., the more expert in the field), the lower the intrinsic cognitive load (complexity) experienced for a given problem”.^68^

Descriptive comparisons based on participants' familiarity with VR did not reveal consistent or substantial impact on participants' overall experience of the inCarne prototype. In particular, no clear differences across user experience or perceived cognitive load dimensions were clearly identified (Table 4). Descriptive comparisons by gender showed broadly similar user experience ratings between female and male participants (Table 4). Given the limited sample size and the exploratory scope of this pilot study, these observations should not be overinterpreted.

### Design implications for embodied VR anatomy tools

Taken together, these exploratory observations suggest that the inCarne prototype may support a broadly accessible and positively perceived visualization experience across different user profiles. This is encouraging from an inclusive design perspective, as it indicates that neither lack of prior VR experience nor differences in anatomy expertise necessarily constitute obstacles to engaging with the system. At the same time, the subgroup patterns observed here remain preliminary. Because this was a pilot study with a small sample, the discussion should remain focused on potential tendencies rather than firm subgroup conclusions. Such work will be important not only to confirm the present tendencies, but also to guide iterative refinement of the system toward inclusive and pedagogically effective immersive learning design.

Concerning the UX design, this study suggests the following implications:

- Focus on maintaining high task efficiency while introducing subtle hedonic elements to enhance the experience without detracting from usability. For example, include cleaner and more professional visuals.
- Add Customization options to streamline workflows (removing unnecessary steps, reducing complexity, and automating repetitive tasks to save time and effort).
- Build on the strong emotional engagement by further improving task efficiency. For example, simplify workflows or provide contextual guidance to make task completion easier or include onboarding tools like tutorials or tooltips to reduce cognitive load.

Beyond reporting user experience and cognitive load outcomes, this pilot study evaluated several concrete design features of the inCarne VR application that may inform future VR anatomy development. First, the integration of real‐time body‐tracked skeletal mirroring without handheld controllers supported embodied interaction. Participants physically executed functional anatomy movements while observing a corresponding skeletal representation. This design emphasizes movement‐to‐content congruence, which may support intuitive understanding of joint mechanics and spatial relations. Second, the system incorporated two complementary spatial reference frames:

1. an egocentric embodied perspective based on body tracking, and
2. an allocentric manipulable perspective using controllers to rotate and inspect the anatomical model.

This frame structure allows learners to alternate between experiencing movement from within their own body and analyzing structure from an external viewpoint. Such spatial flexibility may represent a valuable design principle for functional anatomy tools. Third, the application intentionally employed a minimalist interface structure, aiming to reduce extraneous cognitive load by avoiding unnecessary overlays, menus, or visual clutter. The low extraneous load scores observed in this pilot suggest that limiting interface complexity may be beneficial in immersive learning environments. Fourth, the agile and user‐centered development process enabled iterative refinement based on early UX and cognitive load feedback. Documenting this structured early‐stage evaluation process contributes to the methodological literature on VR educational tool development. Taken together, these features provide transferable design considerations for immersive anatomy applications, particularly regarding embodiment integration, spatial reference management, and cognitive load‐sensitive interface design.

Although this study was conducted within the development framework of the inCarne application, its implications extend beyond the optimization of a single tool. The findings contribute to the broader academic discourse on immersive anatomy education by:

1. Providing descriptive evidence on user experience dimensions and perceived cognitive load in a functional VR anatomy learning context, including embodied anatomical activities. Hence, encouraging early‐stage dissemination and scientific tracing of project methodology and design.
2. Illustrating methodological considerations involved in piloting a VR anatomy learning study using cognitive load and user experience measures in immersive environments.
3. Presenting an example of an agile, user‐centered evaluation process applied during early‐stage development of a VR‐based anatomy learning tool.

These insights may inform future research on immersive anatomy learning environments, particularly regarding instrument selection, embodied interaction design, and early‐stage validation strategies in anatomy educational technology development.

## LIMITATIONS AND IMPLICATIONS

Despite the empirical contribution of this article to identifying user experience factors and correlations, this study entails some limitations. First, the female participation rate and the individual variability within the sample do not reflect the broader social dynamics in our university, which could influence the accuracy of the findings. Second, the data collected were limited to a total of 17 users due to time constraints and participant availability. Thus, no inferential tests could be conducted, and fewer outcomes could confirm potential interactions between dimensions. A further limitation of this study concerns the measurement of cognitive load. Although the Leppink et al.^39^ questionnaire is a widely used instrument, its original validation was conducted in a different disciplinary context and language. The adapted French version used in this study was therefore not fully validated. While intrinsic and germane load items demonstrated acceptable internal consistency, extraneous load items did not, indicating that this dimension was not reliably captured in the present form. As a result, cognitive load findings, particularly those related to extraneous load, should be interpreted with caution. However, this limitation also represents an important outcome of the pilot study, as it informed the need for improved measurement strategies. Future studies will either employ an alternative instrument or conduct further pilot testing to refine and validate extraneous load items specifically for VR‐based anatomy learning contexts.

Further investigation with larger samples is suggested to uncover a more complex and nuanced relationship between user experience potent factors, providing deeper insights about the tested items proposed in this study. The cognitive load measure was a subjective scale administered at the end of the experimental task, thus limiting detection of load variations over time; therefore, it is encouraged to observe the variation of load relative to each task by modern load detectors such as eye tracking techniques (specifically, the frequency of eye blinks). One experience‐limiting aspect is the presence of potential system bugs, as users observed skeleton arms that bend incorrectly, legs that cross improperly, and parasitic tremor of the skeleton at rest. Finally, certain movements were not well realized/detected by the camera's limited precision potential, in particular: prono‐supination, foot movements when aligned with the camera, passing the arm behind the trunk. Future implications suggest increasing the cognitive learning potential of the inCarne tool, by adding ligaments and muscles, and modeling muscle contraction during movements, thus targeting the analysis level on the bloom's taxonomy scale.

## CONCLUSION

In conclusion, this pilot study provides descriptive evidence regarding user experience and perceived cognitive load during interaction with the *inCarne* VR functional anatomy prototype. The findings suggest that the tool was generally perceived positively in terms of user experience and that reported cognitive load levels were within a manageable range for participants. Given the limited sample size and exploratory design, the observed associations between user experience dimensions and cognitive load components should be interpreted cautiously. The primary contribution of this study lies in informing iterative refinement of the application and documenting the methodological considerations involved in early‐stage evaluation of immersive anatomy learning tools. The results provide structured feedback to guide the next development phase of the *inCarne* VR application. Future research will focus on evaluating learning outcomes and conducting larger‐scale validation studies to further assess the educational effectiveness and curricular integration of the tool. Moreover, future studies may further explore how design characteristics relate to perceived cognitive workload and user experience suggesting the following hypothesis: the VR adapted design minimizes cognitive load induced by the difficulty of the learning content and provides a positive user experience; moreover, reducing the cognitive load generated by the VR experience itself can enhance the user experience, thereby enhancing the overall learning experience.

## AUTHOR CONTRIBUTIONS

**Mélanie Gallot:** Writing – review and editing; conceptualization; investigation; supervision; data curation; validation; methodology; formal analysis. **Rawad Chaker:** Writing – review and editing; supervision; project administration; funding acquisition. **Nady Hoyek:** Writing – review and editing; project administration; supervision; funding acquisition. **Mira Hajj‐Hassan:** Writing – original draft; formal analysis. **Christian Collet:** Writing – review and editing; project administration; supervision; funding acquisition.

## Data Availability

The data that support the findings of this study are available from the corresponding author upon reasonable request.
